# Italian health professionals on the mandatory COVID-19 vaccine: An online cross-sectional survey

**DOI:** 10.3389/fpubh.2022.1015090

**Published:** 2022-10-19

**Authors:** Luca Ghirotto, Matías Eduardo Díaz Crescitelli, Ludovica De Panfilis, Luana Caselli, Arianna Serafini, Luca De Fiore, Gian Maria Galeazzi, Massimo Costantini

**Affiliations:** ^1^Qualitative Research Unit, Azienda USL – IRCCS di Reggio Emilia, Reggio Emilia, Italy; ^2^Bioethics Unit, Azienda USL – IRCCS di Reggio Emilia, Reggio Emilia, Italy; ^3^Scientific Directorate, Azienda USL – IRCCS di Reggio Emilia, Reggio Emilia, Italy; ^4^Il Pensiero Scientifico Editore, Roma, Italy; ^5^Department of Biomedical, Metabolic and Neural Sciences, University of Modena and Reggio Emilia, Modena, Italy; ^6^Department of Mental Health and Drug Abuse, Azienda USL – IRCCS di Reggio Emilia, Reggio Emilia, Italy

**Keywords:** COVID-19, vaccine adherence, healthcare professionals, mandatory vaccination, opinions, qualitative survey

## Abstract

Italy was the first country in Europe to make vaccination against COVID-19 mandatory for healthcare professionals by imposing restrictions in cases of non-compliance. This study investigates the opinions of the Italian healthcare professionals' categories affected by the regulation. We performed a qualitative online survey: the questionnaire comprised both close- and open-ended questions. The final dataset included *n* = 4,677 valid responses. Responses to closed-ended questions were analyzed with descriptive statistics. The framework method was applied for analyzing the open-ended questions. The sample spanned all health professions subject to compulsory vaccination, with a prevalence of physicians (43.8%) and nurses (26.3%). The vaccine adhesion before the introduction of the obligation was substantial. 10.4% declared not to have adhered to the vaccination proposal. Thirty-five percent of HPs who opted not to get vaccinated said they experienced consequences related to their choice. The trust in the vaccine seems slightly cracked, demonstrating overall vaccine confidence among professionals. Nonetheless, our results show that whether (or not) professionals adhere to vaccination is not a reliable indicator of consent to how it was achieved. There are criticisms about the lawfulness of the obligation. The data show a great variety of participants interpreting their roles concerning public and individual ethics. The scientific evidence motivates ethics-related decisions—the epidemic of confusing and incorrect information affected professionals. The Law triggered an increased disaffection with the health system and conflicts between professionals. Dealing with the working climate should be a commitment to assume soon.

## Introduction

When the WHO published the policy brief document “COVID-19 and mandatory vaccination: Ethical considerations and caveats” (1), the Italian government had just introduced mandatory vaccination for health professionals (HPs) with Decree No. 44 of April 1^st^, 2021, converted into Law No. 76 of May 28^th^, 2021, which became effective in July (2).

Italy was the first country in Europe to make vaccination against COVID-19 mandatory for this population by imposing restrictions in cases of non-compliance (3). Health professionals who refuse to have the vaccine (uHPs) can be transferred to duties that do not risk spreading the virus or being suspended without pay while the Law is in force, i.e., until December 31^st^, 2022 (2).

The purposes of this law concern the protection of public health, the health system capacity (1), and the prevention of infection. On the one hand, the requirement to work introduced in Italy can be considered morally dutiful, as the immediate implementation of those “mandatory duties” of social solidarity is expressed in Article 2 of the Constitution. On the other hand, it lends itself to being contrary to personal ethics.

As highlighted by WHO, whether a mandate for HPs is proportionate and would not undermine trust might depend on the local context and should be investigated empirically before a mandate is considered for this population (1). Indeed, public trust is essential for vaccination interventions (4). No anticipatory studies or public consultation have been conducted in Italy (unlike in the UK) (5) in an area where worldwide experience in enforcing mandates in the adult population is limited (6, 7). From studies concerning vaccination obligations for the pediatric population, we assume and know best that directives might trigger distrust in the government and increase polarization and antivaccination sentiment (8).

This study investigates the opinions of the HPs categories. It stems from the need to understand their experiences and deepen their perspectives on the critical aspects of the Law and its impact on organizations.

## Materials and methods

We performed a qualitative online survey (9) of HPs in Italy. The questionnaire comprised both close- and open-ended questions.

### Questionnaire development and pilot

In July 2021, we set version 1 of the questionnaire on the Google platform, which was administered to a convenient sample for assessing its comprehensibility. The participants commented on the structure and content of the survey and suggested modifications. After changes had been made, the final version of the questionnaire was composed as follows:

- Questions to collect the sociodemographic and professional information (eight close-ended questions: Q1 gender, Q2 age, Q3 region of employment, Q4 health profession, Q5 work experience—in years, Q6 type of work contract, Q7 work setting, Q8 assistance to Covid patients since March 2020).
- Q9 on the vaccination-related decision (a close-ended question).
  ° Q10 about the intention to receive the vaccine in the future (if the participants decided not to—a close-ended question).
  ° Q11 about whether the decision caused consequences (a close-ended question).
  ° Q12 about the type of consequence (a close-ended question).
  ° Q13 comment on feelings (an open-ended question).
- Questions about opinions on the Law about:
  ° Q14 the obligation by Law (an open-ended mandatory question).
  ° Q15–16 occupational safety (both a close- and an open-ended question).
  ° Q17–18 personal freedom (both a close- and an open-ended question).
  ° Q19–20 protection of public health (both a close- and an open-ended question).
  ° Q21 Penalties stated by the Law (a compulsory open-ended question).
  ° Q22 The motivations underpinning the legislator's choice (an open-ended mandatory question).

### Survey administration

The questionnaire was available online from the 9^th^ to August 31^st^, 2021. The authors disseminated the link to the questionnaire among their professional contacts (also via social networks). They also contacted all the Italian professional organizations. The Italian Order of Doctors, Surgeons, and Orthodontists (FNOMCeO) disseminated the questionnaire to their associates.

### Dataset inclusion criteria

The software used for the survey returned a total of 5,025 respondents. The dataset was then cleaned of duplicate responses, and participants were removed if they did not meet the following inclusion criteria:

- Practicing a health profession recognized by the Italian State as subject to mandatory vaccination.
- Actively working in public or private healthcare structures.

The final dataset included *n* = 4,677 valid responses.

### Data analysis

Responses to closed-ended questions were analyzed using SPSS version 25 (IBM Corp. Released 2017. IBM SPSS Statistics for Windows, Version 25.0. Armonk, NY: IBM Corp.) by LC.

Descriptive statistics were performed for all response variables from close-ended questions, including frequencies and percentages. Contingency tables were produced to explore the joint distribution of responses on vaccine adherence (Q9) and opinions regarding whether mandatory vaccination can ensure safety in the workplace (Q15), respect individual freedom (Q17), and protect public health (Q19).

As to the text analysis from the open-ended questions, we applied the framework method (10), according to an inductive (bottom-up) orientation (the content of the data directs theme development) and a thematic approach (11–13). We followed these steps:

- Each analyst read a selection of responses for each of the open-ended questions.
- Independently, the researchers generated provisional analytical frames by labeling the responses and grouping the labels into themes or meaningful dimensions.
- The frameworks were discussed and compared in teams (LG, LDP, MDC, AS). Disagreements between researchers were resolved through comparison, and a version for each frame was agreed upon.
- The researchers then applied these analytical frames to the remaining data from each open-ended question.
- Any changes made to the frames were discussed and endorsed by the team.

Finally, we compared the analytical frames to obtain the participants' meanings and opinions of the phenomenon as a whole (14). All the authors agreed on the last version of the findings.

### Ethical considerations

The relevant Ethics Committee was approached. It was unnecessary to seek formal approval as the data would have been anonymous at the source, in compliance with current privacy legislation (GDPR—Regulation 2016/679). Nonetheless, participants were informed that, by continuing to fill in the questionnaire, they would consent to data processing by the researchers of the Qualitative Research Unit of the Azienda USL-IRCCS of Reggio Emilia.

## Results

### Study population

The study population entails *n* = 4,677 Italian HPs whose demographic characteristics are shown in Table 1. Sixty-seven percent of the respondents were female, with a median age of 47.3 years (median class: 41–50 years). The sample spanned all health professions subject to compulsory vaccination, with a prevalence of physicians (*n* = 2,049, 43.8%) and nurses (*n* = 1,230, 26.3%). Although with a rather uneven frequency, all the Italian regions as places of employment were represented.

**Table 1:** Demographic characteristics. Total sample (*n* = 4,677).

***Gender***
Women	3,132 (67.0)
Men	1,475 (31.5)
I prefer not to specify	65 (1.4)
Other	5 (0.1)
***Age (years)***	
18–30	389 (8.3)
31–40	866 (18.5)
41–50	1,169 (25.0)
51–60	1,380 (29.5)
>60	873 (18.7)
**Health profession information**
***Health profession areas***
Health professions^**a**^	2,474 (52.9)
Nursing health professions	1,230 (26.3)
Obstetric health professions	79 (1.7)
Rehabilitation health professions	482 (10.3)
Technical health professions	179 (3.8)
Prevention health professions	29 (0.6)
Health operators	204 (4.4)
***Work experience (years)***
0–5	525 (11.2)
6–10	514 (11.0)
11–20	1,077 (23.0)
21–30	1,157 (24.7)
>30	1,404 (30.0)
***Work contract***
Employee	3,291 (70.4)
Freelance	1,021 (21.8)
Trainee	122 (2.6)
Contract	217 (4.6)
Other	26 (0.6)
***Work setting***
Public	3,042 (72.7)
Private	726 (15.5)
Semi-private	549 (11.7)

Data are numbers (%).

^**a**^Physicians, pharmacists, dentists, veterinarians, psychologists, biologists, chemists, physicists.

More than half of the respondents (56.1%) indicated that they had provided care to Covid-19 patients since March 2020. The vaccine adhesion before the obligation was substantial (87%). On the contrary, 10.4% (*n* = 486) declared not to have adhered to the vaccination proposal by choice, and 2.6% (*n* = 120) did not adhere for medical reasons.

The majority of those who decided not to get the vaccination (*n* = 419) confirmed their intention even after the coming into force of the Law; the remaining 67 declared that they would get it because of the obligation.

Thirty-five percent of HPs who opted not to get vaccinated said they experienced consequences related to their choice. The most frequent were the written warning (54.7%) and the suspension from work (26.5%). We summarized vaccine adherence information and consequences at the workplace in Table 2.

**Table 2:** Responses on vaccine adherence and consequences at the workplace.

**Q8. Have you been providing care to COVID-19 patients since March 2020?**	**Total sample**
	**(*n* = 4,677)**
No	1,292 (27.6)
Yes	2,625 (56.1)
Not applicable	760 (16.2)
**Q9. Regarding the SARS-CoV-2 virus vaccine proposed to you as a healthcare professional..**.	
I did it (even a single dose)	4,071 (87.0)
I couldn't do it for medical reasons/I recently had COVID-19	120 (2.6)
I have decided not to	486 (10.4)
**I have decided not to (Q9)**
**(*****n*** **=** **486)**
**Q10. If you have decided not to, what is your intention for the future?**	
I don't mean to	419 (86.2)
I will do it because it is mandatory	67 (13.8)
**Q11. Did this choice have consequences at work and/or actions by your employer/professional register?**	
No	316 (65.0)
Yes	170 (35.0)
**Q12. In case of any consequences, of what kind?**	
Written warning	93 (19.1)
Change of duties	2 (0.4)
Demotion	3 (0.6)
Suspension	45 (9.3)
Other	27 (5.6)

Data are numbers (%).

### Qualitative findings

By employing the framework method, we grouped open-ended questions' data into the following areas: the interpretation of the legislator's motivations, the obligation by Law, occupational safety and public health, the respect for individual freedom, and the penalties. The response rates to close-ended questions and participants' percentages, shown in Table 3, also enrich the thematic areas.

**Table 3:** Responses of vaccinated vs. unvaccinated healthcare professionals on mandatory vaccination (Q9) and occupational safety (Q15), individual freedom (Q17), and public health (Q19).

	**Vaccine adherence (according to responses to Q9)**	
	**Yes (*n* = 4,071)**	**No (*n* = 606)**
		**-For medical reasons**
		**-By choice**
**Q15. In your opinion, is mandatory vaccination helpful for making your workplace safer for everyone?**		
Yes	3,705 (91.0)	25 (4.1)
		-22
		-3
No	265 (6.5)	544 (89.8)
		-92
		-452
Other	101 (2.5)	37 (6.1)
		-6
		-31
**Q17. In your opinion, is mandatory vaccination respectful of your freedom?**		
Yes	3,333 (81.9)	34 (5.6)
		-17
		-17
No	738 (18.1)	572 (94.4)
		-103
		-469
**Q19. In your opinion, is mandatory vaccination helpful for protecting public health?**		
Yes	3,773 (92.7)	40 (6.6)
		-26
		-14
No	298 (7.3)	566 (93.4)
		-94
		-472

Data are numbers (%).

#### What are the legislator's motivations according to HPs?

About 60% of vaccinated HPs (vHPs) felt that the legislator promulgated the obligation, first and foremost, out of concern for HPs and patients. The legislature would also have been worried about the socio-economic implications for the health system and the country. Secondly, the legislator would have introduced the obligation for HPs to increase the total number of vaccinated people (as even some uHPs thought) while compensating for the number of those who did not vaccinate (21%). The Law would have given a strong message to doctors and neutralized the low intellectual level of many grossly uninformed HPs. For the 2%, the Law aimed to strengthen the concept of science, particularly by emphasizing the correct channel of scientific information.

Almost 46% of uHPs (and 10% of vHPs) felt that the passing of the Law was due to electoral reasons and the government's subservience to the pharmaceutical industry. It was the opinion of the uHPs that the Italian government wanted to make propaganda, exercising a policy of fear to maintain power. For the 17% of uHPs, the legislator wanted to exploit the ease of obliging a specific professional category. It made the HPs vaccine's “testimonials,” forcing them to become an example for the population. The Law also acted as an acceptability test for the vaccine to be imposed on the whole population (also using the “green pass” strategy). The leaders would use this legislative strategy as a “coercive warning” to counteract hesitancy.

The underpinning motivation of the Law was achieving purposes against people for one-third of the uHPs. Some respondents (11%) stated that the legislator would intentionally pursue social control, restrict citizens' freedoms, eliminate non-aligned HPs, implement large-scale scientific experimentation, and the “new world order.” Many respondents felt they were “guinea pigs” in this respect.

For some HPs, the mandate would be set to forcibly counter the uncertainty of the available evidence, i.e., a defensive approach, giving everyone the illusion of control over the pandemic (10% of uHPs) or providing a simple answer to a complex problem (5% of vHPs).

#### The law

Approximately 25% of vHPs considered the mandate by Law justified on ethical grounds. Accepting the obligation would be a human duty and in line with the deontological aspects of the medical and health professions. Some stated that professional responsibilities include diagnosis, treatment, and prevention: mandatory vaccination should be interpreted in the latter sense as a commitment to protect others, especially the most fragile and vulnerable patients. It was also specified as an ethical value to preserving the functioning of the health system (20%). However, 6% referred to emerging organizational problems if the uHPs were suspended.

On the other hand, about 50% of uHPs and 15% of the vHPs stated that the Law would be unconstitutional, undemocratic, and illegitimate. It would violate self-determination's constitutional principles (as in a dictatorial regime).

Furthermore, for the 34% of uHPs, the Law would be unethical due to the co-occurrence of two conditions: the experimental nature of the treatment (which would not have completed the experimental process) and the coercion to sign an informed consent (an ethically hypocritical act). The 7% of these participants felt abused and forced to do something unacceptable because it was considered risky for their health, not supported by unambiguous scientific evidence.

In both groups of participants (4% of vHPs and 17% of uHPs), we identified the theme of the ambivalence of the scientific knowledge possessed. In this dimension, a bipolarity of positions is evident: among vHPs, some respondents used references to scientific data or studies to support the vaccination requirement, while others, at the same time, expressed doubts and perplexity, to the point of considering the norm a “duty” with no scientific basis. For uHPs, on the other hand, opinion is homogeneous in considering the vaccination requirement inconsistent with medical and scientific knowledge. All these respondents felt that their scientific knowledge and competencies were disregarded: the vHPs by the uHPs, the non-vaccinated by their superiors, and the working environment, in which, some stated, there would be no place and no right to exist for doubt. In response, for vHPs, imposing the requirement was necessary to increase vaccine uptake among HPs and counteract the doubtfulness of colleagues.

#### Safety and public health

Most vHPs saw the Law as a valuable strategy to increase and ensure occupational safety (91%) and public health (92%), as shown in Table 3. Working with vaccinated colleagues and other staff would provide security and trust to ease tensions and create a relaxed atmosphere. For the 50% of vHPs, the vaccine would be the only way forward in public health as there is a lack of other equally effective strategies. Compulsory vaccination would therefore be a way to protect public health as it would protect those who cannot vaccinate and the good functioning of the national health service.

Some, however, did not agree, and the reasons overlapped with the uHPs' opinions. The obligation would not increase safety (89.8%) or public health protection (93.4%) as swabs and antibody counts did. For the 12.4% of vHps, the obligation would push vaccinated personnel to lower their alertness threshold and, therefore, not to use personal protection equipment (PPE) properly, no longer complying with safety standards to prevent contagion. In addition, swabs and PPE would be more protective of the public, whereas the vaccine would only safeguard the vaccinated individual (37.6% of vHPs). In this sense, 43.9% of uHPs repeatedly stressed how vHPs could infect their colleagues and patients equally. In addition, there was a consensus among uHPs that the obligation was superfluous because healthcare workplaces were already safe after the reorganization of services after the first wave (26.6%).

For the 25.3% of uHPs, the vaccine would threaten public health: forcing people to be vaccinated would increase the emergence of variants.

#### Freedom

Table 3 summarizes that most vHPs believed that the Law respects individual freedom (81.9%). On the contrary, almost all uHPs stated the opposite (94.4%), showing an intense polarization of opinions. Half of the vHPs (52.5%) noted that the issue of respect for personal freedom was not at all in question since vaccination would be a requirement for working in healthcare, in line with the Hippocratic Oath. For the 27.5% of vHPs, individual freedom would be a secondary value for the common good and civic duty.

However, some vHPs (19.9%) considered that the Law undermines individual freedom. In this, they agree with uHPs. For the 53% of uHPs, the regulation would be coercion, irreconcilable with the democratic values of the country, antithetical to the Constitution, and, therefore, an expression of a Nazi-fascist regime. For the 46.2% of vHPs, this directive would sanction an obligation not to think, choose, or exercise freedom of care for oneself. Every medical act should, on the contrary, be a choice to be made with one's doctor and not by the imposition of the employer. The obligation to vaccinate was not considered consistent with other individual freedoms (e.g., abortion and gender reassignment) that healthcare companies support.

#### Penalties

18.3% of vHPs stated that sanctions would be an inevitable step in enforcing the Law. There was substantial agreement among respondents. However, many vHPs (13.8%) opposed applying sanctions, which they considered abuse and an attack on labor rights. In contrast, 58.7% of vHPs stated that sanctions should be punitive and increased to dismissal, disbarment from professional registers, and imprisonment. For vHPs, sanctions would be an incentive not only to vaccinate (some respondents stated that they got vaccinated because of the sanctions) but also to understand the scientific value of the data available and, therefore, to respect science and its methods.

On the contrary, for the 78% of uHPs, sanctions would be an act of force, humiliating, and even psychologically, a blow to working dignity. In this sense, sanctions would be disproportionate and inappropriate measures would cause increased discontent among HPs (10.2%). Those uHPs who stated that they suffered repercussions on their professional and personal life due to the implementation of the Law generally indicated that they had the feeling of living under blackmail, of feeling a victim of mobbing, discrimination, with related negative emotions (27.4%). 16.8% of uHPs reported feeling angry, others afraid of both economic and relational consequences that their choice caused. In this context, 37% of these participants stated how the obligation and the effects they experienced reinforced their beliefs.

## Discussion

Reliance on vaccination seems slightly cracked, demonstrating overall vaccine confidence among HPs (15). At the time of the survey administration (August 2021), 1.82% of the total HPs working in Italy were without a single dose (16), with 94.42% having completed the vaccination cycle. Mandatory vaccination for HPs in Italy had a positive result, which was doubted to be obtained in the UK, where the potential success of a mandatory vaccination policy for HPs was questioned. The UK Government estimated that only a minority of current uHPs would be vaccinated under the policy, leaving 5% of the workforce unvaccinated (17). In France, where vaccination was not compulsory for HPs, HPs' intention to get vaccinated against COVID-19 varied across time and professional categories. On May 2021, the French public health agency reported that 91.7% of HPs had received one dose, and 63.4% were fully vaccinated (18).

It is widely accepted that mandatory vaccination for HPs could obtain high coverage and increase its uptake (18, 19). With the efficacy of such intervention beyond the scope of this study, our qualitative results show that whether (or not) HPs adhere to vaccination is not a reliable indicator of consent to how it was achieved. Even among vHPs, there are criticisms about the lawfulness of the obligation. Therefore, the adhesion rate is not directly proportional to the rate of understanding or approval of the obligation. This confirms that vaccine mandates are effective (20) while remaining controversial (21, 22).

Having this in mind is fundamental for policymakers and healthcare authorities when proposing immunization by Law. All immunization programs should feature a combination of mandatory and voluntary instruments (23). However, the fact that a consistent fraction of vHPs and not only of uHPs reported a high level of perceived coercion suggests that vaccination enforcement should be accompanied by more effective efforts at explanation and persuasion (24). Furthermore, as noted in the French context, a mandatory vaccination policy should be associated with HPs' adequate training, including and targeting all concerns (18). Investigating initial HPs' concerns, fears, and beliefs about the COVID-19 vaccine would have been desirable in all the countries where it has become a mandate. Our data reveals that feelings of coerciveness stemmed from concerns not voiced and heard by the policymakers.

This study highlights that each HPs' beliefs and opinions shape some voluntary elements. The data show the variety of participants interpreting their roles concerning public and individual ethics. In a social-cultural context where HPs are expected to indubitably adhere to vaccinations, considered standard requirements like hand washing (25), HPs are likely to feel pressed to undergo the vaccination (26, 27) as a moral imperative (25). In this situation, autonomy is perceived to be eroded, leaving ethical issues not completely fulfilled (18) and public/professional and individual ethics subject to be questioned.

It has been suggested that a successful strategy for policymakers to increase vaccination rates is nudging HPs to make better decisions by offering encouragement which considers personal barriers preventing expected choices from taking place (21). “Nudges incentivize vaccinations and help better align vaccination intentions with near-term actions” (21).

In this context, policymakers and healthcare authorities are encouraged to address with HPs the issue of balancing moral compromise and public health prevention. Besides, the relationships between individual rights and the role of the State as guarantor of the collective good (relying on available scientific evidence) seem to necessitate a reconfiguration and a wider acceptance.

Our results show that what motivates ethics-related decisions is also the personal understanding of what scientific evidence means. Therefore, it is legitimate to wonder about HPs' level of knowledge of medical science and its methodological aspects (28). A great deal of confusion about what a drug testing process means, and entails emerged. We noted in the data how ‘science' had been used for opposing purposes, how data was read based on personal bias, and how evidence-based was both trustworthy and a source of false information. Scientific misconduct and conflicts of interest detected over time may have damaged the level of trust and prevented an informed and correct understanding of science and its results. Acknowledging past abuses and their effects on evaluating subsequent interventions could be the base for transparent and honest scientific communication and re-education.

Concomitantly, the epidemic of confusing and incorrect information also affected HPs. The considerable amount and plurality of information circulating during this period have now frightened, now confirmed, now confused HPs. Further research on how evidence dissemination was performed among HPs and understanding the consequences of evidence dissemination methods is desirable.

As to impacts on organizations, the Law triggered, for some, increased disaffection with the health system for which respondents work. Conversely, it started a perception of an increase in conflict between professionals. Dealing with the working climate seems a commitment that Italian healthcare authorities should assume soon. As shown elsewhere (29), interpersonal conflict consequences include perceptions of a disrespectful working environment and weakened team collaboration, which negatively affect the quality of care. In this context, while eliminating the coercion related to the obligation is not impossible, it is certainly possible to address stigmatization, isolation, and devaluation messages for those who doubt and dissent, which could lead to an improvement in the working climate and containment of the negative psychological consequences related to obligation.

This survey was carried out on a large, varied sample, representing all the different health professions involved in the Italian Law, which, even after the rules for containment have been relaxed for the general population, remains in force for HPs. This study aimed to understand and systematically organize the opinions of HPs on the core elements of the vaccine obligation by Law.

This study did not aim to understand the reasons for vaccine hesitancy after the Law. Nevertheless, many of the answers to the questions on compulsory vaccination were related to the vaccine. Respondents have often (mostly unconsciously) substituted the questions on the obligation with questions on the vaccine, on which they have a more definite opinion.

On COVID-19 vaccine hesitancy among Italian HPs, an extensive survey conducted in October 2020 reports that 67% (*n* = 1,155) of HPs would take COVID-19 vaccination, with 7% declaring their refusal (30). The survey's authors explain that among the reasons for hesitancy, there was a lack of trust in the safety and efficacy of vaccines.

When vaccines are considered safe (for instance, childhood vaccines against poliomyelitis, diphtheria, tetanus, pertussis, hepatitis B, measles, mumps, and rubella), Italian HPs tend to agree with vaccination through by-Law obligation (31). However, our results demonstrate that for HPs, childhood vaccines are considered significantly different from the COVID-19 vaccines, whose safety and efficacy were doubted, even in August 2021, months after the start of the Italian vaccination campaign. These same concerns explained the low acceptance rate of HPs in other countries (where HPs' vaccination is not mandatory) (32, 33), where two-thirds declared they were contrary to a COVID-19 compulsory vaccination policy (33).

The legislation on vaccines is generally conditioned by the validity of the results of medical-scientific research, in constant evolution, on the safety and efficacy of vaccines (16). Nonetheless, HPs' opinions and beliefs play a core role in the acceptance and consent. A cross-sectional study (within the UK-REACH study https://uk-reach.org/main/) on HPs' views on mandatory SARS-CoV-2 vaccination in the UK highlights how it is vital that substantial efforts are made to build confidence in the safety and efficacy of vaccines among those who are hesitant (17). This suggestion aligns with what was also reported by a scoping review about COVID-19 vaccination hesitancy in HPs, mapping evidence worldwide (34). Building vaccine confidence among HPs would likely not just increase uptake or enable HPs to advocate for vaccination among patients but also avoid augmented mistrust towards public healthcare interventions and policies.

Further overlaps between the Italian survey conducted in October 2020 (30) and our study are interesting to note: they regard the doubtfulness related to the hurried experimental process and the low trust in pharmaceutical companies and control authorities. Those concerns being stable display that the introduction of mandatory policies nurtures ethical issues and impairs trust in authorities (8, 17, 19, 20, 30) and does not improve the rate of understanding or approval of the obligation.

In general, the survey highlighted several areas worthy of work on to understand adherence to the Law and its impact on individuals, society, and organizations. This study provides potentially helpful evidence to contextualize the vaccination obligation in Italy and the current political and social fallout. They also concur to give a picture of the level of adherence and acceptability for the measure of compulsory vaccination compared to the softer measure of vaccination by recommendation. Lessons learned from this mandatory vaccination could be helpful to other public health programs and suggest carefully considering “unintended consequences” (20). Given our findings, we agree with Bardosh et al. (20) when they suggest that mandating vaccination should be used cautiously; it may trigger distrust, raise ethical issues, and deteriorate the work climate. We advocate for a continuous re-evaluation of COVID-19 vaccine policies, especially in Italy, where leveraging empowering strategies based on trust and public consultation were missed and are still missing.

## Study strengths and limitations

This is the first study to consider the role of HPs' opinions and beliefs about the COVID-19 vaccine legislation. This survey intercepted a heartfelt topic. By using a qualitative approach, our study could map all the meaningful dimensions around the core aspects of the Law. Participation in the qualitative survey, which featured many open-ended and challenging questions, was considerable. Our recruitment strategy reached many HPs across all the categories and Italian regions. In addition, surprisingly, we collected opinions from many uHPs, whose views enriched the dataset. Nonetheless, selection biases and the limit of a convenient sampling method (35) are noteworthy and affect the statistical representativeness of the findings and the possibility of conducting a logistic regression to examine any associations between the demographic characteristics of participants and their opinions about mandatory vaccination.

## Data availability statement

The raw data supporting the conclusions of this article will be made available by the authors, without undue reservation.

## Ethics statement

Ethical review and approval was not required for the study on human participants in accordance with the local legislation and institutional requirements. The patients/participants provided their written informed consent to participate in this study.

## Author contributions

LG, LDP, and MC: conceptualization and writing—original draft preparation. LG, MC, LC, MD, GG, and AS: methodology, formal analysis, and investigation. LDF, LDP, MC, GG, and LC: writing—review and editing. LG: supervision. All authors contributed to the article and approved the submitted version.

## Conflict of interest

The authors declare that the research was conducted in the absence of any commercial or financial relationships that could be construed as a potential conflict of interest.

## Publisher's note

All claims expressed in this article are solely those of the authors and do not necessarily represent those of their affiliated organizations, or those of the publisher, the editors and the reviewers. Any product that may be evaluated in this article, or claim that may be made by its manufacturer, is not guaranteed or endorsed by the publisher.
